# Towards renewed commitment to prevent maternal mortality and morbidity: learning from 30 years of maternal health priorities

**DOI:** 10.1080/26410397.2023.2174245

**Published:** 2023-03-01

**Authors:** Andrea Solnes Miltenburg, Birgit Kvernflaten, Tarek Meguid, Johanne Sundby

**Affiliations:** a Associate Professor in Global Health, Department of Community Medicine and Global Health, Institute of Health and Society, University of Oslo, Oslo, Norway; Resident in Obstetrics and Gynaecology, Department of Obstetrics and Gynaecology, Akershus University Hospital, Lørenskog, Norway.; b Researcher, Department for Interdisciplinary Health Sciences, Institute of Health and Society, University of Oslo, Oslo, Norway; c Associate Professor, Consultant Obstetrician & Gynaecologist, Department of Maternal and Child Health, University of Namibia, Windhoek, Namibia; d Professor, Department of Community Medicine and Global Health, Institute of Health and Society, University of Oslo, Oslo, Norway

**Keywords:** Safe Motherhood, maternal health, SDG, quality of care, women’s needs

## Introduction

The Sustainable Development Goals (SDGs) created a renewed commitment to improve maternal health in low-income countries. The estimated number of women worldwide dying in childbirth reduced from 532,000 in 1990 to 303,000 in 2015.^1^ However, maternal health remains highly inequitable between and within populations, as the burden of complications falls disproportionately on the most vulnerable in areas where health systems are the weakest.^2^ Increased awareness of the existing implementation gaps of global strategies illustrates that global attention on maternal health does not necessarily result in sufficient action in the places where it is needed most.^3^ Additionally, the last three decades focused on Safe Motherhood have shown a pervasive global promotion of narrowly framed indicators and interventions, which has resulted in neglect of the social, economic, and political determinants that contribute to poor maternal health. In this article, we reflect on the global maternal health strategies of the past 30 years (1987–2017). Through the concept of “travelling models”,^4^ we aim to show how *models of care* have been framed and prioritised as global standards and have subsequently travelled to various low-resource settings. We explore what impact they have had on local practices, and finally what lessons can be learned to inform the priorities of the current decade.

## Travelling models of care

Global strategies and recommendations for maternal health are designed based on “easy to measure” indicators and focus on evaluating progress toward predetermined goals to reduce maternal deaths. As a result, maternal health interventions have been modified, multiplied, and refined based on emerging evidence and knowledge of “what works”.^5^ Olivier de Sardan et al.^4^ apply the concept of “travelling models” to their study of maternal health care and argue that global strategies and policies are often highly standardised, exported from one setting to another in an almost identical format across numerous country contexts. They argue that policies and protocols are based on “miracle mechanisms” that are taken out of their original context and believed to be as successful in other settings. Models thus refer to standardised intervention programmes, including recommendations, guidelines, and interventions, aimed at producing social change through changes in the behaviour of one or more categories of actors,^4^ and in the present case, that follow from global perspectives of an ideal “model of care” for pregnancy and childbirth.

The theory of change and dissemination approach of the ideal model of care has changed slowly over the past three decades (see Table 1) towards an increasingly medicalised and technocratic approach to pregnancy and childbirth. Examples of this include: the steady increase of caesarean sections, the centralisation of maternity hospitals, and extensive high-tech diagnostics (e.g. ultrasound, fetal monitoring, prenatal diagnostics). This has occurred because of historical analysis of what works to reduce maternal deaths in high-resource settings, mixed with what is perceived to be realistic in resource-constrained settings.^6^ Koblinsky et al.^6^ described four models of care, differentiated according to the organisational characteristics of where women give birth, who provides care, and what services this care includes. Models one to four (see Table 1) represent stages in the medicalisation of maternity care, but do not necessarily imply a value hierarchy. When reviewing the last 30 years, different models of care representing each decade have travelled in an almost identical format as a “one-size-fits-all” to many countries. The promoted model of care within low-resource settings has progressed from Model 1 in the first decade to Model 4 in the third decade. This affected where women were recommended to give birth, who was expected to be present and what services should be provided. Currently, women in many resource-poor settings worldwide are encouraged to give birth in facilities, in a setting with access to a skilled birth attendant (SBA) and comprehensive emergency obstetric and new-born care (EmONC), even though in reality these services might be absent, inaccessible, unaffordable, or of poor quality for many.

**Table 1.** Travelling models in three decades of maternal health priority setting.

	Priority interventions	Primary outcome effect	Models of care	Theory of change	Where
Decade I (1987–1997)	• ANC – risk identification	Mortality reduction	*Model 1*: (Home birth with lay provider)	Early recognition and identification of risk leads to prevention and timely management of complications.	Community / Primary Care Level (health centre)
	• Training of TBA		*Model 2:* (Home birth with a skilled provider)		
Decade II (1997–2007)	• SBA at primary care level (health centre)	Mortality reduction	*Model 3:* (Facility birth with SBA and access to EmOC)	Skilled attendance at birth in a health centre or hospital with early identification and referral through emergency transport in case of complications to EmOC facility.	Facility / SBA at primary care level, EmOC Referral level
	• Emergency transport				
	• EmOC at referral facility				
Decade III (2007–2017)	• ANC – focused	Mortality reduction	*Model 4:* (Facility birth with SBA in EmONC facility and access to CEmONC)	ANC as a place to provide interventions proven to be effective in reducing mortality and promoting health-seeking behaviour for birth. Birth attended by providers skilled to manage complications in a setting with access to CEmOC.	Facility / all levels
	• SBA and EmONC at primary care level				
	• CEmONC at referral level				

Abbreviations: ANC: antenatal care; TBA: Traditional Birth Attendant; SBA: Skilled Birth Attendant; EmOC: Emergency Obstetric Care; CEmOC: Comprehensive Emergency Obstetric Care; EmONC: Emergency Obstetric and Newborn Care; CEmONC: Comprehensive Emergency Obstetric and Newborn Care.

Global strategies shaped far away from the realities and contexts in which they are implemented, may limit the state’s ability to govern and develop its own national health priorities and health system.^7,8^ At the same time, such global policies and interventions are moulded in the local context and managed by local actors. The models of care are understood, negotiated, rejected, or circumvented by local health workers and women who are the targets of these interventions.^4^ In the following section, we show how a global standard model of care has been framed and prioritised and subsequently travelled to various local settings. We use country examples to illustrate the impact of these somewhat radical changes that have occurred. Writing the full history of maternal health strategies is beyond the scope of this Perspective and we acknowledge that the process of making policies has been filled with controversies, debates, and disagreements.^9,10^

## Three decades of action

### The first decade (1987–1997): putting maternal mortality on the global agenda

When the Global Safe Motherhood Initiative (SMI) was launched in 1987, estimations showed that approximately 500,000 women worldwide died each year due to pregnancy and childbirth-related causes, affecting mostly poor and vulnerable women in low-income countries.^11^ As a coordinating mechanism as well as a body to develop policy recommendations and lead on advocacy initiatives, the SMI issued a specific goal to reduce maternal mortality by 50% by the year 2000, situating maternal health within the context of improving women’s status in economic, social, and political spheres.^12^ The Maternal Mortality Ratio (MMR) became the primary indicator by which to assess progress, although this indicator was challenged due to the lack of appropriate definitions, unclear classification of causes of death, and lack of surveillance and reporting systems in the countries where most women were dying. Alternative methods of measurement (e.g. the sisterhood method) and sophisticated models of estimation and approximation were thought to strengthen the reliability of the MMR.^13^ The World Health Organization (WHO) started to provide global estimates – with different methodology of measurement – for each country.

The SMI’s first decade (1987–1997) successfully placed the problem of maternal mortality on the global agenda, resulting in several conferences on Safe Motherhood. In addition to improving women’s status and educating communities, conference participants in Nairobi in 1987 argued that immediate beneficial results would be achieved by focusing on concrete health sector interventions. These interventions included: (1) strengthening community-based health care, (2) improving referral facilities, and (3) developing alarm and transport systems.^12^ However, in practice, key actors and the public health community prioritised two specific interventions: antenatal care (with a focus on screening for women at high risk) and training of traditional birth attendants (TBAs), to improve care at birth.^14^ This was based on the reality that most women were not reached by a professional midwife or obstetrician as births mainly took place at home (Model 1). By the end of the first decade, there was an increasing need to move from advocacy to action, which called for more information on country, state, and global mortality figures, measurement of impact of interventions, efficiency of resource use, and evaluation of progress.^15^

### The second decade (1997–2007): evidence-based prioritisation of interventions

The Safe Motherhood Technical Consultation in 1997 reviewed the available evidence and articulated a consensus on priority action messages for the second decade,^16^ which clearly pointed to the need for a comprehensive multi-sectoral approach to maternal health. Yet the most influential statement to emerge from this consultation was, “having a health worker with midwifery skills present at childbirth, backed-up by transport in case emergency referral if required, is perhaps the most critical intervention for making motherhood safer”.^16^ Two interventions were thus put forward: EmOC* and SBA. Evaluation of the evidence of the effectiveness of risk screening during antenatal care and training of TBAs showed little effect on reducing maternal mortality. For these interventions to be effective, this would require a functional health system, including quality obstetric care and a functioning referral system.^17,18^ As a result of these new priorities, many donors and governments began to de-emphasise, or even ban, training of TBAs and instead prioritised increased access to professional medical care, especially for life-threatening complications.^17^

The launch of the Millennium Development Goals (MDGs) in the year 2000 situated maternal health and reduction of maternal mortality (MDG 5) as central to poverty reduction and overall development. This increased international attention on maternal health and the SMI.^19,20^ SBA, access to basic and comprehensive EmOC, and effective transport systems were the three essential interventions promoted under the MDG 5 indicator of “skilled birth attendance”, which served as a proxy for the MMR.^21^ The shift from antenatal to intrapartum care, as well as the shift from TBA to SBA, was justified by the observation that most deaths occur around the time of birth.^22^ Therefore, the professionalisation of birth care (Model 3) became the new hope to generate renewed commitments to reducing maternal mortality by institutions and governments.^23^

### The third decade (2007–2017): continuum of care

The third Safe Motherhood decade (2007–2017) began with the use of phrases such as, “getting on with what works”, “stripping away the complexities”, and “simplification of the issues”.^5^ Comprehensive became equivalent to “packages” of evidence-based single interventions that were seen to be effective in tackling maternal mortality along the continuum of care.^24,25^ Emphasis remained on the MDG Countdown to 2015 Agenda, aiming to achieve a reduction of MMR of 75% (indicator 5.1). In this decade, antenatal care received renewed interest through promotion of the Focused Antenatal Care model, which includes a minimum of four visits with a specifically determined number of single evidence-based interventions.^26^ The evidence for this model was based on a large multicentre randomised controlled trial, which reduced the number of necessary visits from “many” to four, in normal pregnancies. However, the trial did not include low-income countries, where one or two visits were the norm.^27^

Skilled attendance at birth (MDG Indicator 5.2) continued to be promoted strongly as a priority solution,^5,28^ although there were considerable challenges in measurement. It remained far from clear which providers were considered skilled and which single actions this strategy included,^9,29,30^ consequently most studies were only able to perform progress assessments on the number of facility births, without clarity on what services women received and from whom. Effectiveness of the SBA strategy alone to reduce mortality was therefore limited and ultimately was dependent on the accessibility and availability of “back-up” referral care.^29,31^ In contrast, the EmONC strategy and the seven signal functions were backed by an increasing evidence base, although there was still limited research in low-income settings.^32^ With Comprehensive EmONC in place it was estimated that most deaths, including maternal deaths, new-born deaths, and stillbirths could be averted.^32,33^ Consequently, the message to prioritise Comprehensive EmONC was clear and simple, and directly targeted the time when most deaths occur. Focusing on all births taking place in an EmONC facility with, at minimum, close proximity to a Comprehensive EmONC facility (Model 4) was thought to allow for quick results and thus would appeal to donors and governments.^34^

## Travelled models in health system crisis

### Where, by whom, and what care is delivered

When Koblinsky and colleagues presented the different models of care in their publication in 1999, they focused specifically on ensuring that implementation of these models was adapted to the capabilities of the local health systems.^6^ The authors warned against making the transition from promoting home birth (Model 1) to hospital birth (Models 3 and 4) too quickly, before adequate human resources, equipment, and supplies were available to secure good quality of care. Nevertheless, the emphasis on facility-based birth, which was strongly advocated for in most low-income countries in the second decade (1997–2007), resulted in a quick shift from Model 1 to Model 3. This was largely a consequence of the pressure to perform well according to the Countdown to 2015 Agenda aiming to increase the facility birth target. “Global goals were converted into national planning targets” and therefore became the driving force for national health policy planning.^10^ In some local settings, implementation of new care models resulted in the use of coercion or financial incentives pushing women to give birth in health facilities, with disregard for contextual differences regarding availability and accessibility of care (see Box 1).

**Box 1 Country examples**

**India:** In India, the government introduced conditional cash transfer programmes, a financial incentive scheme to increase the level of institutional births in India among the poor. Mothers living below the poverty line receive payments, as does the female health worker responsible for bringing the mothers to a public health facility to give birth.^35^ Public health facilities, that have become overburdened, lack an enabling environment and sufficient resources to ensure quality of care.^36^ “Skilled attendance” in hospitals turned out to be the attendance of sweepers and ward boys.^35^ Also, informal payments or requests to purchase medicines and other supplies continued to be observed, in particular among recipients of the cash transfer programmes, who were generally the most vulnerable population.^37^

**Malawi:** In Malawi, a radical policy shift included official banning of TBAs and fining them for supporting women during home birth. Women and their husbands were fined a goat or a small amount of money if they gave birth at home. Despite successful promotion of the new policy, its local implementation had not been accompanied with an increase in health personnel and supplies, resulting in mistrust between health facilities, women, and local leaders with regard to what the money from fines was used for.^38^ Overburdened health facilities resulted in a reduction in the quality of care and caused women to give birth unattended. A re-introduction of user fees led to further deterioration in women’s access to services.^39^

**Nicaragua:** In Nicaragua, massive pressure for facility-based delivery banned TBAs from attending births. Pregnant women were monitored, followed, and brought into health facilities to prevent maternal deaths.^40^ Women desiring home births were reluctant and expressed concerns about inattention and neglect from hospital personnel and assessed these health facilities as incapable of providing good and appropriate care.^40,41^ In response to women preferring home births despite the government emphasising facility births, public health officials referred to these women as ignorant and troublesome. Forced implementation of the policy disregarded the social, cultural, and economic accessibility for the most marginalised populations, engendering resistance to facility-based birth, and making women hide their pregnancies from local health facilities,^41^ which may have contributed to delays in care-seeking, even in the case of complications.

**Tanzania:** In Tanzania, the government had initially planned to train 32,000 TBAs by the year 2000. A few years later, TBAs were banned from assisting at births and their training ceased throughout Tanzania.^42^ Investments focused instead on training SBAs and ensuring the availability of EmOC. This led to a highly inequitable health system, with unequal distribution of resources and concentration of services in urban facilities, leaving women in rural areas severely disadvantaged.^43^ In order to increase the availability of SBAs in rural areas, attempts were made to increase the number of cadres of health workers (e.g. clinical officers). Lack of resources was a continuing challenge and poor quality of care led women to delay care-seeking or to bypass lower-level health facilities, contributing to overcrowding at referral hospitals.^44^

What the country examples have in common is that inequity, poverty, inequality, and inadequate access to services are widespread and that the health system struggles to keep up with targets that aim to achieve high levels of facility-based births. Additionally, an over-emphasis on the ill-defined SBA led to rapid training of multi-purpose health workers in specific skills areas (in particular EmONC), resulting in an influx of birth attendants who are not truly skilled in the full range of midwifery care and lack the essential resources to do their work.^45^ Ultimately, this has left some countries with a cadre of unsupported TBAs and a limited number of insufficiently trained SBAs, without investments in the surrounding health system, as well as formal and informal health systems that are disconnected from each other.

### The issue of quality of care

Towards the end of the MDG era, progress evaluations revealed that despite increasing availability of, and access to, skilled care at birth, including EmONC, the MMR in many countries did not reduce as expected. A “mismatch between burden and coverage” exposed “a crucial gap in the quality of care”.^46^ Quality of care is multi-faceted and complex and can be judged based on several dimensions, including the outcome of care (mortality and morbidity), provision of care (coverage of evidence-based practices for routine care and complications), or the experience of care by women and their families.^47^ Prioritisation of care for pregnant women in low-resource settings has focused on the technical aspects of care, notably basic and comprehensive EmONC, because this has the greatest potential to reduce maternal mortality at the population level. The travelling “models of care” resulted in simplifying the intervention focus and have steered resources away from the community, and away from peripheral health facilities where women still lack basic, safe, hygienic, and respectful birthing environments. A focus on EmONC has also led to a neglect of the important role of midwives in improving health outcomes for women and new-borns.^48^

Not only is it important to consider *where, by whom, and what care* is delivered. But also, *how* this is delivered. It is at the individual provider level where the concept of quality of care is most meaningful.^49^ Insufficiently trained personnel and lack of essential medicines and supplies exacerbate poor quality of care. Chronic staff shortages, increasing numbers of women that are seeking care, increasing expectations of performance, and an increasing number of interventions are severely overburdening health workers.^50^ Amongst the economic, social, and cultural conditions that constrain women’s use of health services, poor quality of care, unequal power relations between health providers and women, and different views of the pregnant and birthing body influence women’s expectations and experiences of care.^51^ Attention to the “blind-spot” of disrespect and abuse in childbirth, led to a strong call to confront the harsh local realities of resource-constrained settings and the lack of value given to “what women need and want”.^52^

### The human rights approach

In many ways, it was hoped that the human rights approach would revive the original aims of the SMI,^23^ namely, failure to address maternal mortality and morbidity, as a result of injustices and the cumulative denial of women’s human rights.^53^ During the time of the MDGs, largely because of the political climate, rights perspectives on maternal health were more or less ignored and only became marginally included in 2005 when goal 5B was added, aiming for “universal access to reproductive health”.^53^ It wasn’t until 2012, when the UN adopted a ground-breaking resolution regarding a human rights-based approach in the context of Safe Motherhood that it was firmly established that “states have a human rights obligation to guarantee women of all racial and economic backgrounds, timely and non-discriminatory access to appropriate maternal health services”.^54^ The practical implementation of the human rights approach still faces numerous obstacles. As a result of gross social inequalities in many countries with high maternal mortality, the ability of individuals to claim their rights and hold people or institutions accountable remains severely compromised.^55^

In Brazil, the case of Alyne da Silva Pimentel Teixeira versus the government was the first time a national government was held accountable for a maternal death. Her case is of significance because not only had the government failed to ensure appropriate services, but the ruling also focused on the violations which occurred in ensuring quality of care, with reference to both provision of sub-standard care according to clinical guidelines, as well as the neglect and discrimination contributing to her death.^56^ In 2011, a charter for Respectful Maternity Care (RMC) was developed. This followed an increase in publications detailing women’s experiences of disrespect and abuse during facility-based childbirth.^57^ The recent WHO guidelines specifically focus on the importance of RMC where women’s values and needs are central. However, the practical implications of these new guidelines face obstacles on the ground. Many facilities in low-resource settings harbour a culture of dehumanised care where disrespect and abuse have been normalised.^58^ Several studies point out that drivers of mistreatment of women during childbirth, beyond health system factors, include broader social and gender norms that facilitate abuse.^59^ Addressing disrespect and abuse through policies, guidelines, and interventions that “travel” across the globe, are unlikely to change fundamental drivers in society that maintain women’s inferior status, especially if these are not context-specific and adapted to local culture and norms.

## A call to action for the fourth decade

The SDGs promote a broader perspective and acknowledgement of diversity, and go beyond mortality to consider morbidity, disability, and functionality – moving beyond physical health to include social and mental well-being.^60^ As a contrast to narrow indicators, the SDGs conclude that multi-sectoral approaches offer potential by acknowledging the broader roots of vulnerability, such as gender discrimination, and placing emphasis on important infrastructural interventions, such as roads, water, and sanitation. We anticipate the travelling model in the fourth decade of maternal health to include a priority focus on “quality of care”, through a model of care that is context-specific and a primary outcome effect of “maternal well-being”. This means we are moving away from a preoccupation with maternal death, and thus from using mere survival as a measurement of progress.^13,23^ The question remains how this current momentum can result in adequate action at the country and facility level to ensure women’s needs are truly met instead of continuing with “business as usual”.

We offer three lessons on how to move forward: (1) Comprehensive multifactorial approaches are not prioritised when “simple” concrete interventions offer an alternative, even if these use poorly defined indicators. (2) The move towards an increasingly medicalised model of care requires careful planning and consideration of the existing services, providers, skills, and desires of women. (3) Coercion and forceful implementation of interventions reduce trust between women, families, communities, providers, systems, and policy makers which will take time to repair.

Key interventions to reduce maternal mortality include complementary, mutually reinforcing strategies.^61^ The SMI and later the Partnership for Maternal, Newborn and Child Health (PMNCH) advocated for such comprehensive strategies for three decades. Yet the models that travelled across the globe and that were prioritised were based on narrow indicators, often poorly defined and resulting in interventions with sometimes insufficient evidence of effectiveness in resource-poor settings. Donor-driven funding approaches flourishing after the MDGs play a major role in this.^62^ The quality of care indicators as presented by the WHO,^47^ although broadened and based on a growing evidence base, remain a simplified approach towards a complex problem. When “travelling” to local contexts, indicators do not function as anticipated.^4^ In striving to reflect a more reliable picture of reality, the main indicator against which progress was measured over the years, changed from MMR to SBA to Facility Birth, often through the use of countrywide demographic and health surveys. Limitations of relying on such data are now widely acknowledged.^63^ Relying on only quantitative measurements neglects the implementation process and unexpected contextual challenges that occur. There is a need for greater emphasis on other research methods to explore how known interventions can be effective at country, district, and facility levels. Such understanding can only be obtained by performing qualitative studies. Much is still needed to increase the use of such studies for decision-making.

The standardised and increasingly medicalised models of care implemented in many low-income countries manifest differently depending on the available health system, as well as political, cultural, and social factors. For that reason, countries adapt and use tactics to strive for quick results. Based on the knowledge we have today of reducing maternal mortality, policy makers should identify solutions that could be more easily adapted to each country’s existing health system and context. Available health providers (formal and non-formal) should be included, learning from the different skills they have (both relevant biomedical and social/cultural skills) and depending on what women and communities desire. Examples from Malaysia and Sri Lanka show how an incremental and pragmatic approach to ensuring equitable access to good quality care, underpinned by strong political support and elimination of financial barriers, can lead to changes over time.^6,34^ In Bangladesh, success was dependent on a pluralistic health system, use of several care models, and pursuing women-centred, gender-equity-oriented health programmes, through the work of widely deployed community health workers reaching all households.^64^ Ultimately, for many countries this will mean going back to the basics and building simple systems that work, which can be quite different from one region to the next. Travelling models of care should therefore be more context-sensitive and allow for understanding of local and regional constraints, resources, aspirations, norms, and strategies.^4^ There are examples from several local settings that focusing on locally available solutions can work.^65,66^ If women’s values and needs are the true driving force in this current decade of maternal health priorities, the minimum action required is to ensure that no woman is alone during birth, that a birth companion is present, and that the birth environment allows for respect for individual women’s dignity.^67^ It is fundamental that women, their families, health providers, and managers on the ground collectively determine what is required to achieve this and which models of care fit best in the given context. This inevitably means that health providers’ needs are met as well. Working conditions with lack of safe water and basic sanitation, lack of essential resources and support systems can greatly increase feelings of demoralisation^68^ and risk for burnout among providers.^50^ This naturally limits their ability to provide quality of care and should not be neglected. Investments made in previous decades to push for facility birth may have caused damage to women and health providers on the ground. Mistrust between institutions and communities likely influences the situation of today.^14,69^ It is time to focus on healing and repairing trust, which will only be possible if we are able to create the political will to invest in high-quality, respectful care. We need to finally acknowledge that valuing women and improving quality means letting their voices be heard and putting funds and time not just into improving the health systems and models of care we have now, but into substantially changing them.

By the time of publication, we are well into the fourth decade, and efforts towards progress or change have been severely disrupted, and perhaps even reversed, by the COVID-19 pandemic.^70^ The pandemic clearly showed how a one-size-fits-all approach resurfaced and had clear drawbacks. Lockdowns and curfews had their place in many high income settings, but were problematic, impossible, or disastrous in many low-income settings.^71^ Global focus on numbers of deaths due to COVID-19 overshadowed a fuller picture of local realities. Pregnant women suffered greatly from the decreasing access to health services and deteriorating quality of care. Recent estimates show that this has resulted in a reduction in health service use and increase in maternal and new-born mortality and morbidity.^70^ However, major investments in containment strategies for COVID-19 such as improvements in overall testing infrastructure, patient flow and hand washing policies, alongside increasing awareness of the importance of context-specific interventions, likely mitigated the effects of the pandemic in some settings.^72^ If maintained, lessons learned from these mitigation strategies can be of benefit for women and their families.

## Conclusion

The emphasis on global goals and targets that travelled across the globe during the past 30 years successfully reduced the number of maternal deaths, but has failed to address the concerns that are so important for the most vulnerable, including issues of access, affordability, and quality. It is therefore worth reiterating that reflecting on thirty years of Safe Motherhood calls for some humility^69^ and recognition of the role the global community has played in maintaining the status quo for many women in low-income settings. We should acknowledge the limitations of travelling models, that inevitably will continue to influence care in low- and middle-income countries, and instead provide for “context-sensitive” implementation.^4^ This also means allowing for sufficient time for changes to occur. There is no time like the present, to translate lessons learned into actions that truly reflect women’s needs in the places where they live and give birth.

## Footnotes

* We focus on the term EmOC rather than EmONC in the first two decades because the “N” for Newborn was not included in the defianition until the third decade, through initiatives such as the Every Newborn Action Plan, UNICEF 2013.
